# Unusual Presentation of Elastofibroma Dorsi on ^18^F-FDG-PET/CT: A Case Report

**DOI:** 10.1097/MD.0000000000002832

**Published:** 2016-02-18

**Authors:** Steve P. Martin, Joanna Gariani, Claire Tabouret Viaud

**Affiliations:** From the Department of Imaging and Medical Information Sciences, Radiology (SPM, JG); and Department of Imaging and Medical Information Sciences, Nuclear Medicine (CTV), Geneva University Hospital, Geneva, Switzerland.

## Abstract

A 70-year-old male patient underwent an Fluorodeoxyglucose-positron emission tomography-computed tomography for staging of a left parahilar lung neoplasm found during work-up for fatigue and asthenia. The scan demonstrated a hypermetabolic lung tumor, a hypermetabolic pleural effusion and 4 hypermetabolic bilateral soft tissue lesions of the chest wall corresponding to 4 elastofibroma dorsi. Initially, the oncologic disease was classified as stage IV because of the hypermetabolic pleural effusion. A transbronchial biopsy showed squamous cell carcinoma and the cytology of the pleural effusion revealed no malignant cells. As the other 4 hypermetabolic thoracic wall lesions were correctly diagnosed as benign despite their unusual presentation, the patient underwent surgery by left pneumonectomy and mediastinal lymphadenectomy. The lymph node involvement required adjuvant chemotherapy.

Diagnostic confidence of the benignity of the hypermetabolic chest wall lesions allowed a more aggressive treatment with a better outcome after a malignant pleural effusion was excluded.

## INTRODUCTION

Positron emission tomography/computed tomography (PET/CT) is used in daily practice, particularly for oncological staging. Hypermetabolic foci must be correctly interpreted for accurate staging. In our case, the hypermetabolic pleural effusion and the hypermetabolic chest wall lesions could modify staging, as these could translate secondary tumor locations. The chest wall lesions were atypical for elastofibroma dorsi because of their increased number and unusual Fluorodeoxyglucose (^18^F-FDG) uptake. This atypical presentation must be recognized to avoid erroneous staging.

## CASE REPORT

**FIGURE 1:**
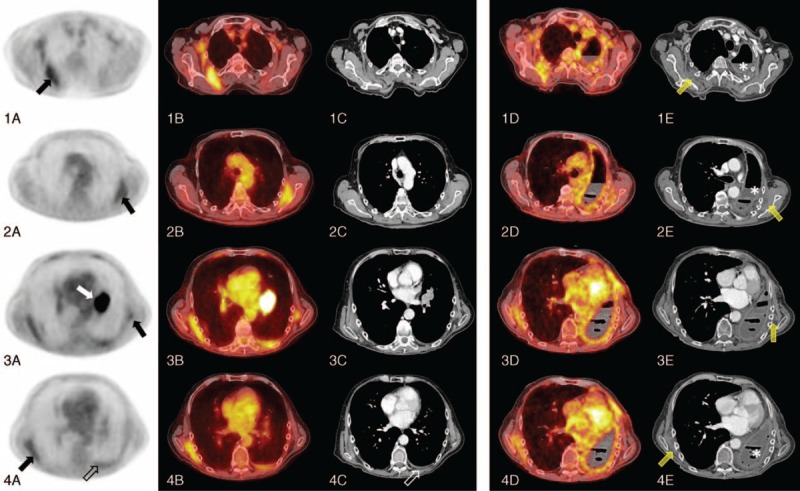
Axial images of an Fluorodeoxyglucose-positron emission tomography-computed tomography (^18^F-FDG-PET) scan performed on a Siemens Biograph mCT PET scanner 60 minutes after administration of 190 MBq ^18^F-FDG (1–4A); axial fusion images of the ^18^F-FDG-PET scan and a free-breathing non-contrast-enhanced computed tomography (1–4B); axial images of a breath hold intravenous iodine contrast computed tomography scan in mediastinal windowing (1–4C). Three-month follow-up axial fusion images of a ^18^F-FDG-PET scan and a free-breathing non-contrast-enhanced computed tomography (1–4D). Three-month follow-up axial images of a breath hold intravenous iodine contrast computed tomography scan in mediastinal windowing (1–4E).

**FIGURE 2:**
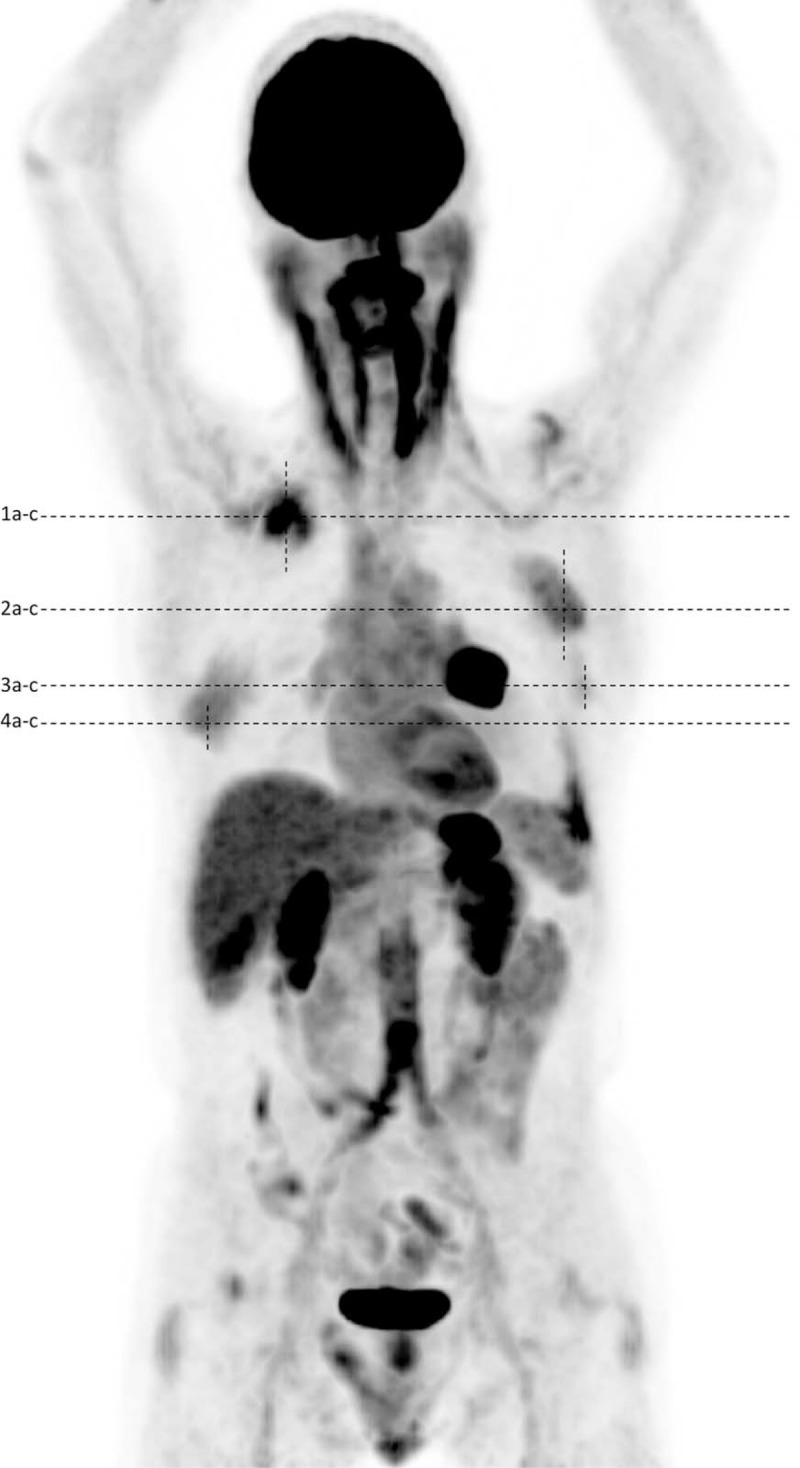
MIP image of the Fluorodeoxyglucose-positron emission tomography-computed tomography (^18^F-FDG-PET) acquisition. The highest SUV_max_ measured on elastofibromas dorsi was 4.7 (Fig. 1 1images 1a-c). This image also shows an intense uptake of an aortoiliac stent 16 years after surgery, probably because of an infection.

A 70-year-old male patient presented with progressive asthenia, involuntary weight loss, and decreased appetite. A CT scan showed a left parahilar mass. The patient was then referred to our imaging department for staging by ^18^F-FDG-PET/CT. The multimodal examination identified the known primary lung neoplasia as a hypermetabolic left parahilar mass (maximum standardized uptake value [SUV_max_] 18), an associated hypermetabolic left pleural effusion (SUV_max_ 6) and 4 hypermetabolic bilateral soft tissue masses of the posterior chest wall with an increased ^18^F-FDG uptake up to SUV_max_ 4.7 for the most hypermetabolic mass measuring 5 × 1.6 cm and located under the right latissimus dorsi muscle (Figure 1 image 1B). The left upper lesion measured 5.8 × 1.5 cm with a SUV_max_ 3.8 (Figure 1 image 2B) and the left lower lesion measured 2.2 × 1.1 with a SUV_max_ 2.1 (Figure 1 image 3B). The right lower lesion measured 5.2 × 1.4 cm with SUV_max_ 3.2 (Figure 1 image 4B). All lesions presented similar morphologic characteristics; they were ill-defined lesions of soft tissue density with fat stranding. The selected images demonstrate 4 hypermetabolic foci (black arrows, Figures 1 and 2) Fig. 1, 2) corresponding to elastofibromas dorsi. The primary lung lesion (white arrow, Figure 1) corresponds to the hypermetabolic left parahilar mass (Figure 1, images 3A, B, C). A sixth hypermetabolic focus (empty arrow) is seen (Figure 1 images 4A, B, C) and was suspicious of a carcinomatous pleural effusion. Because of the hypermetabolic pleural effusion, the disease was initially diagnosed as stage IV.

The transbronchial biopsy showed a squamous cell carcinoma and the cytology of the pleural effusion revealed no malignant cells. According to these new findings, the patient underwent video-assisted thoracoscopy surgery (VATS) for intrapericardial left pneumonectomy. The pathological diagnosis confirmed the type of lung cancer, complete resection, and demonstrated intrapulmonary lymph node involvement. The hypermetabolic pleural effusion was not of carcinomatous origin and was probably linked to an infectious left lung base consolidation. The final Tumor Node Metastasis (TNM) classification was pT2a N1 M0 R0, stage IIa. The high risk of recurrence owing to lymph node involvement required adjuvant chemotherapy.

A 3-month post-surgery follow-up by ^18^F-FDG-PET/CT showed an unchanged aspect of the 4 elastofibroma dorsi (yellow arrows, Figure 1 images 1-4D, E), post-operative changes with left pneumonectomy (asterisks, Figure 1 images 1–4D, E) and no suspect hypermetabolic lesions.

## DISCUSSION

Elastofibroma is a benign poorly circumscribed soft tissue lesion classically located in the subscapular region, hence the denomination elastofibroma dorsi. This entity can also be found in other less frequent locations such as the olecranon,^1^ the thighs,^2^ or less often, in subcutaneous tissue.^3,1^ Its histopathological characteristic consists of an accumulation of abnormal elastic fibers.^4^ The prevalence of such findings on computed tomography (CT) scans is of 2% in the elderly population with a clear female preponderance (F:M ratio 13:1), although some recent studies reported a prevalence ranging from 13% to 17% and even up to 81% in some autopsy studies.^5,6^ Elastofibroma dorsi are frequently asymptomatic and unilateral but bilateral involvement is described in 10% to 66% of cases.^4,7^ Even though a case of 17 elastofibromas has been reported,^3^ multiple elastofibromas are rare. When symptomatic, patients report tumefaction, mild pain, and a clicking sensation.^8^ Surgical resection is the treatment of choice when elastofibromas are symptomatic or >5 cm in diameter.^7^ Correct diagnosis of this benign pseudotumor prevents excessive resection as the risk of recurrence is very low with marginal excision.^4^

Imaging features of elastofibroma dorsi include lack of a capsule, poor differentiation with surrounding muscles, soft tissue density, heterogeneity with strands of fat, and absence of bone abnormalities.^9,10,11^ Magnetic resonance imaging (MRI) is often considered the modality of choice due to excellent soft tissue resolution; however, findings may remain nonspecific. Nonetheless, CT seems to be more accurate in determining the size of elastofibroma dorsi.^6^ These lesions may be difficult to detect based solely on their morphology, as in our case. The initial CT had not retained any chest wall lesions and it was their metabolic activity on the PET/CT that revealed them.

According to different studies, the mean SUV_max_ (±standard deviation) ranges from 1.4 to 3.2^12^ or is measured at 2.0 ± 0.63 (range 0–5.1)^11^ or at 2.31 ± 0.61 (range 1.0–4.30) (10).

In an oncological context, the differential diagnosis includes metastases and other primary lesions such as fibromatosis, fibrolipoma, desmoid tumors, or sarcoma.^8^

Our case is atypical owing to the sex of the patient, the number of lesions, and the slightly increased ^18^FDG uptake. However, the number of lesions and their slightly unusual uptake should not lower the diagnostic confidence based on the typical CT appearance and location.
